# Fibroblast Growth Factor Inhibitors for Treating Locally Advanced/Metastatic Bladder Urothelial Carcinomas via Dual Targeting of Tumor-Specific Oncogenic Signaling and the Tumor Immune Microenvironment

**DOI:** 10.3390/ijms22179526

**Published:** 2021-09-02

**Authors:** Hye Won Lee, Ho Kyung Seo

**Affiliations:** 1 Department of Urology, Center for Urologic Cancer, National Cancer Center, Goyang 10408, Korea; nsproper@naver.com; 2 Division of Tumor Immunology, Department of Cancer Biomedical Science, Research Institute, Graduate School of Cancer Science and Policy, National Cancer Center, Goyang 10408, Korea

**Keywords:** urothelial bladder carcinoma, precision medicine, fibroblast growth factor receptor, fibroblast growth factor inhibitor, tumor microenvironment, treatment resistance, immune checkpoint inhibitors, combination

## Abstract

Locally advanced or metastatic urothelial bladder cancer (a/m UBC) is currently treated using platinum-based combination chemotherapy. Immune checkpoint inhibitors (ICIs) are the preferred second-line treatment options for cisplatin-eligible a/m UBC patients and as first-line options in cisplatin-ineligible settings. However, the response rates for ICI monotherapy are modest (~20%), which necessitates the exploration of alternative strategies. Dysregulated activation of fibroblast growth factor receptor (FGFR) signaling enhances tumor proliferation, survival, invasion, angiogenesis, and immune evasion. The recent U.S. Food and Drug Administration approval of erdafitinib and the emergence of other potent and selective FGFR inhibitors (FGFRis) have shifted the treatment paradigm for patients with a/m UBC harboring actionable FGFR2 or FGFR3 genomic alterations, who often have a minimal-to-modest response to ICIs. FGFRi–ICI combinations are therefore worth exploring, and their preliminary response rates and safety profiles are promising. In the present review, we summarize the impact of altered FGFR signaling on a/m UBC tumor evolution, the clinical development of FGFRis, the rationale for FGFRi–ICI combinations, current trials, and prospective research directions.

## 1. Introduction

Patients with non-muscle invasive urothelial bladder cancer (NMI-UBC, carcinoma in situ, Ta, or T1), which accounts for approximately 75% of initial UBC diagnoses, demonstrate unexpectedly high recurrence rate and multifocality with disease progression to muscle-invasive UBC (MI-UBC), which has a much less favorable prognosis and occurs in 10–15% of patients diagnosed with NMI-UBC [1,2,3,4,5,6]. For patients who present with non-metastatic MI-UBC, consensus guidelines recommend radical cystectomy and urinary diversion combined with lymph node dissection following cisplatin-based neoadjuvant chemotherapy. However, according to the available scientific data, 50% of patients with MI-UBC develop distant metastasis despite radical cystectomy, and 5% of UBC patients are present with metastasis at diagnosis. Although approximately 50–70% of locally advanced or metastatic UBCs (a/m UBCs) patients respond to chemotherapy, unfortunately, in most cases, progression or recurrence occurs with conventional strategies, and limited benefit is seen in second-line and later setting [2,3,4,5]. The prognosis of patients affected by locally advanced or metastatic (a/m) UBC remains dismal, with a 5-year overall survival (OS) of approximately 10–15% [1,2,3,4,5,6].

Recently, the efficacy of immunotherapy using immune checkpoint inhibitors (ICIs) has been investigated in patients with a/m UBC [1,4,5,7,8]. Anti-programmed death-1 (PD-1) agents pembrolizumab and nivolumab, as well as anti-programmed death ligand-1 (PD-L1) agents avelumab and atezolizumab, have been approved by the USA Food and Drug Administration (FDA) for treating a/m UBC patients who do not respond to platinum-based chemotherapy and have demonstrated durable clinical benefits with reduced toxicity. However, only a subset of patients may respond to ICIs (objective response rate (ORR): 15–21%), and treatment options are limited for patients who do not respond to ICIs. For such patients, antibody-drug conjugates (ADCs) and targeted therapies/anti-angiogenesis agents, which are still under clinical trials, remain the only viable treatment strategies, while taxane-based or vinflunine chemotherapy has modest results but is still used in clinical practice [2,4,5,8].

Multi-platform, high-throughput next-generation sequencing (NGS) technology has enabled comprehensive assessment of the UBC landscape and significantly improved our understanding of its complex pathology, ushering in a new era of precision oncology [2,4,5,8,9,10]. Advances in genomic profiling, the development of targeted therapies, and the resurgence of ICI have led to the molecular subclassification of a/m UBC, and efforts are underway to define therapeutic strategies and associated predictive biomarkers. Receptor tyrosine kinases (RTKs), which transduce extracellular signals to a variety of intracellular signaling cascades [11,12], are classified into the epidermal growth factor receptor (EGFR) group (EGFR, HER2, MET, and RYK, among others), the fibroblast growth factor receptor (FGFR) group (FGFRs, colony-stimulating factor 1 receptor (CSF-1R) and vascular endothelial growth factor (VEGF) R2, among others.), the insulin receptor (INSR) group (INSR, insulin-like growth factor 1 receptor (IGF1R), ALK, and ROS1, among others), the RAR-related orphan receptor (ROR) group (ROR1, ROR2, DDR2, and NTRK1, among others), and the EPH receptor (EPH) group (EPHA1, EPHB1, and PTK7, among others) [8,11,12,13,14,15,16]. The human FGFR family includes four highly conserved RTKs: FGFR1, FGFR2, FGFR3, and FGFR4, which are encoded by distinct genes.

Gain-of-function coding mutations, gene fusion, and gene amplification are three major classes of FGFR alterations associated with the luminal-papillary subtype of a/m UBC [4,5,8,15,17,18]. In spite of the general association between FGFR alterations and favorable characteristics in NMI-UBC, there is no evidence to suggest that FGFR gene alterations correlate with a less aggressive phenotype once urothelial carcinoma advances. In fact, FGFR3 gene alterations are associated with less favorable outcomes in the context of chemotherapy for a/m UBC [15,17,19]. Erdafitinib, a tyrosine kinase inhibitor (TKI) of FGFR1–4, has shown significant benefits in patients with a/m UBC with FGFR alterations [20,21]. In the present review, we summarize the current understanding of the oncogenic signaling of FGFR alterations in a/m UBC, the therapeutic implications of FGFR inhibitors (FGFRis) based on the mode of action of tumor cell and tumor microenvironment (TME) modulators and the accumulated experience to date of using FGFRi–ICI combination therapy. We have focused on the mechanistic differences of FGFRis and ICIs, emphasizing their synergistic efficacy and tolerability compared to monotherapies. Advances in our understanding of a/m UBC biology, coupled with large-scale gene expression and sequencing results, have led to more clinically favorable targeted treatments and effective immunotherapies. The identification and validation of targets and potential biomarkers for predicting the response will be crucial for successfully incorporating novel therapeutic strategies in the evolving landscape of a/m UBC treatment.

## 2. Genomic Alterations Associated with Aberrant FGFR Signaling Activation in a/m UBC

**Figure 1:**
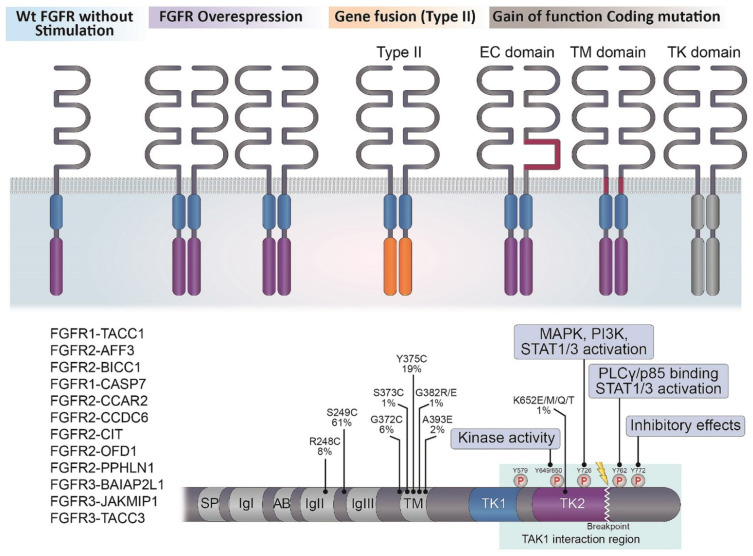
The mechanisms of activated FGFR signaling activity in a/m UBC. The canonical and endocrine FGFs produce their biological actions by signaling through FGFR1-4, which are consisted of three extracellular (EC) parts, a transmembrane domain (TM) and two intracellular (IC) tyrosine kinase domains (TK1 and TK2). Wt = wild type.

**Figure 2:**
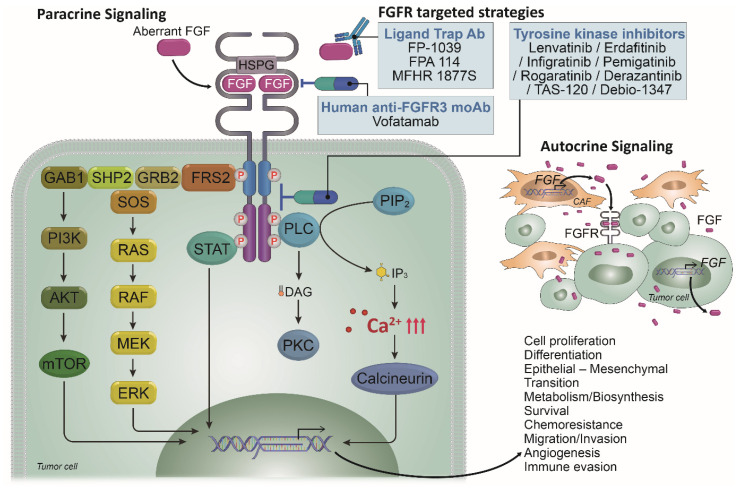
Multifaceted roles of FGFR signaling pathways in a/m UBC and therapeutic strategies. In conjunction with heparin sulfate proteoglycan (HSPG), the receptors bind FGF ligands, leading to receptor dimerization and autophosphorylation and the specific phosphorylation site can bind and phosphorylate substrate proteins to activate multiple signal transduction pathways. FGFR dimerization, kinase activation and trans-autophosphorylation lead to context-dependent activation of downstream signaling pathways. FGFRi reduces phosphorylation of FGFRs themselves and their direct targets, FRS2 and PLC-γ, and inactivate downstream signaling, such as the RAS-ERK, PI3K-AKT, IP3-Ca^2+^ and DAG-PKC signaling cascades. Red upper arrow = an increase in cytosolic concentration.

The canonical and endocrine FGFs exert their biological effects by signaling via FGFR1-4, which consists of three extracellular (EC) domains, a transmembrane (TM) domain, and two intracellular tyrosine kinase (TK) domains (TK1 and TK2) [4,5,8,15,17,22,23,24,25,26] (Figure 1). The EC region encompasses three immunoglobulin-like subdomains (I, II, and III) and an acid box, which is typically located between subdomains I and II, whereas the FGF ligand-binding site is located on subdomains II and III. The TM region is made up of a single α-helix, and the IC tyrosine kinase domain exhibits the canonical bilobed architecture of the protein kinases. In conjunction with heparin sulfate proteoglycan (HSPG), the receptors bind FGF ligands, leading to receptor dimerization and autophosphorylation, and each specific phosphorylation site can bind and phosphorylate substrates to activate multiple signal transduction pathways (Figure 2) [4,5,8,11,13,15,16,17,22,23,24,25,26].

Exquisitely precise fine-tuning of FGFR activity occurs via multiple steps of splicing and regulated expression, activity, and downstream signaling [11,13,16,17,23,25,26,27]. FGFR1-3 generates two additional major splice variants of the Ig-like domain III, referred to as IIIb and IIIc, which are concerned with ligand-binding specificity [16,26,27,28,29]. The receptors and their isoforms are expressed in a cell- and tissue-specific manner to perform specific roles in different tissues and at different stages of development. Consistently, FGFR dimerization, kinase activation, and trans-autophosphorylation lead to context-dependent activation of downstream signaling pathways (Figure 2). Upon ligand activation, the FGFRs dimerize and TK domains become phosphorylated and engage with various downstream proteins, such as FGFR substrate 2 (FRS2) and phospholipase C γ (PLCγ), as well as diverse transduction pathways, such as RAS-MAPK, PI3K/AKT, inositol-1,4,5-trisphosphate (IP3)–Ca^2+^, diacylglycerol (DAG)–protein kinase C (PKC), and Janus kinase (JAK)-STAT [11,13,16,17,23,25,26,27]. As the downregulation of the activated receptors is important to prevent dysregulated signaling, a defective FGFR ubiquitination system and/or an error in the mitigation pathway could induce aberrant cell growth and malignant transformation.

Unlike other receptor tyrosine kinases (RTKs), such as EGFR and vascular endothelial growth factor (VEGFR), in which activating mutations tend to occur exclusively within the kinase domain, mutations in FGFR1–4 have been reported in the EC domain, the TM domain, and the IC TK domain (Figure 1) [11,13,16,17,23,24,25,26,27]. Somatic gain-of-function mutations in FGFR1–4 can cause the receptor to be constitutively active by inducing increased dimerization, enhanced kinase activity, or enhanced affinity for FGF ligands. Somatic activating mutations of FGFR2 and FGFR3 are more common than those of FGFR1. FGFR3 mutations commonly occur in the EC (R248C, S249C) and TM (G370C, Y373C) domains and the cysteine residues encoded by these mutations lead to ligand-independent dimerization of the receptor in a/m UBCs [17,24,26]. Activating FGFR3 mutations are identified in less than 15–25% of MI-UBC cases.

*FGFR* fusion mutations occur via chromosomal rearrangement or translocation and lead to increased receptor dimerization and activation, as well as the dysregulated expression of FGFR or its fusion partner gene (Figure 1) [17,24,25,26,30,31]. A majority of FGFR fusion mutations occur in-frame to produce a functional chimeric protein, which can be categorized as type I or type II depending on whether the N or C terminus of FGFR is involved in the rearrangement, respectively [24,30,31,32]. Both types of FGFR fusion proteins are endowed with oncogenic potential through the acquisition of protein–protein interaction modules from fusion partners for ligand-independent dimerization and/or recruitment of aberrant substrates. Fusions involving FGFR2/FGFR3 and transforming acidic coiled-coil containing protein 3 (TACC3) are the most commonly detected fusion events, followed by fusions involving nucleophosmin 1 (NPM1), TACC2, and bicaudal c homolog 1 (BICC1), which bring about receptor oligomerization and activate one of the FGFR kDs. For example, FGFR3-TACC3 in a/m UBC can phosphorylate the phosphopeptide peptidylprolyl cis/trans isomerase NIMA-interacting 4 (PIN4) by activating the mitochondria and subsequently promoting mitochondrial respiration, de novo sterol and lipid biosynthesis, metabolism, and tumor growth, eventually triggering the RAS/MAPK and JAK-STAT signaling pathways [17,24,33]. Interestingly, the last exon of FGFR3, which is lost in all fusions identified in UBC, includes Y762, which is implicated in PLCγ activation and p85 binding and is a part of a region (amino acids 589–806) involved in interactions with and phosphorylation of transforming growth factor-β-activated kinase 1 (TAK1) [17,24,33]. Interactions with TAK1, and its phosphorylation, lead to the activation of NF-κB [17,24,33]. Thus, it is predicted that downstream signaling activated by these fusions will differ from that of intact FGFR3 [17,24,33].

The ligand-dependent signaling triggered by FGFs derived from cancer cells and stromal/immune cells in the TME plays a key role in the a/m UBC evolution (Figure 2) [11,13,17,23,24,25,26,30]. In addition, although FGFR3 protein is barely detectable by immunohistochemistry in normal urothelium, the upregulated expression has been detected in several UBC tissue samples of all grades and stages [11,17,24]. The expression of FGFR3-targeting miRNAs, including miR-99a and -100, is downregulated in UBC. FGFR3 fusion transcripts lack the 5′ UTR of FGFR3, which contains recognition sites for regulatory miRNAs, leading to the upregulated expression [11,17,24,30,34]. Transcription factors implicated in FGFR3 regulation include hypoxic inducible factor (HIF)-1α, which induces FGFR3 upregulation under hypoxic conditions [11,17,24,30,34]. Rearrangements in the distal enhancer region, as well as point mutations in the proximal promoter region, can induce FGFR overexpression [11,24,30,34]. Collectively, ATP-dependent BRG1/BRM-associated factor (BAF), mutation in chromatin remodeling complex SWI/SNF that dysregulates chromatin remodeling, and the cancer-associated transcription factors also result in FGFR3 overexpression [11,35,36].

## 3. FGFRis in a/m UBC Act as a Dual Modulator of Tumor Cells and the TME

To better appreciate the role of FGF/FGFR signaling during a/m UBC progression, its contribution to the functional interplay among the key players within the TME must be unraveled [11,17,23,24,25,26,30]. The TME compromises the function and the fate of tumor-infiltrating immune cells by creating a three-dimensional structure favoring immunological tolerance and reducing the antitumor efficacy of immunotherapeutic intervention. The TME consists of both cancer cells and stromal/immune cells, such as cancer-associated fibroblasts (CAFs), endothelial cells, lymphocytes, M2-type tumor-associating macrophages (M2-TAMs), myeloid-derived suppressor cells (MDSCs), and neutrophils [23,24,25,26]. Thus, dual targeting of tumor cells and the tumor-promoting TME may exert synergistic antitumor effects and delay the development of drug resistance [11,17,23,24,25,26,30,37]. Combination therapy based on regulating the TME for sensitizing drug activity and decreasing dosage is currently under investigation.

FGFRis reduce phosphorylation of FGFRs directly and indirectly via their targets, FRS2 and PLC-γ, and inactivate downstream signaling via RAS-ERK, PI3K-AKT, IP3-Ca2+, and DAG-PKC signaling cascades [4,5,8,17,23,24,25,26,30,38,39,40,41,42]. In the TME of a/m UBC, the luminal-papillary subtype of the consensus classification is characterized by a high rate of FGFR3 mutations and translocations, suggesting that these tumors may respond to FGFRi [4,5,8,15,17,18,37]. Moreover, the FGFR3 pathway is activated in non-T-cell-inflamed tumors, which are likely to be intrinsically resistant to ICIs. FGFRi elicits antitumor effects directly in cancer cells by suppressing tumor cell survival, epithelial–mesenchymal transition (EMT), invasion, metastasis, and the development of treatment resistance, as well as indirectly through the normalization of the TME, especially paracrine signaling, angiogenesis, and immune evasion (Figure 2) [4,5,8,11,15,17,18,23,24,25,26,30,37,38,39,40,41,42,43,44,45].

## 4. Monotherapy FGFR-Targeting Strategies for a/m UBC

As the role of FGF-FGFR signaling in a/m UBC has become clearer, a large number of potential and promising drugs targeting this signaling pathway have been developed. According to their mode of action, they can be divided into three categories: (a) small-molecule FGFR TKIs (non-selective and selective), (b) anti-FGFR antibodies, and (c) FGF ligand traps and DNA/RNA aptamers [4,5,8,11,15,17,23,24,25,26,30,37,38,39,40,41,42,43,44,45] (Figure 2, Table 1). As the FGFR TKIs may target other growth factor receptors because the binding pocket of ATP-competitive FGFRs shares a high degree of homology with other oncogenic RTKs, such as VEGFR and platelet-derived growth factor receptor (PDGFR), these TKIs can be divided into multi-kinase (non-selective) FGFRis and FGFR-specific TKIs (selective) [23,24,25,26,30,43].

### 4.1. Non-Selective Small Molecule FGFRis

Initial clinical FGFR inhibition studies used non-selective FGFR TKIs, including dovitinib (TKI258), Brivanib (BMS-540125), nintedanib (BIBF1120), and lenvatinib (E7080), which, although not designed to target FGFR specifically, can reversibly and competitively bind to, and therefore disrupt, the ATP-binding pocket in FGFR1–4 [23,24,25,26,30,43]. For example, dovitinib exerts inhibitory effects against FGFR1–3, VEGFR1–3, PDGFR-β, Fms Related Receptor Tyrosine Kinase 3 (FLT3), and macrophage colony-stimulating factor-1 (CSF-1), as these receptors are related phylogenetically and are highly homologous [24,43]. Importantly, VEGF-VEGFR2 and FGF2-FGFR1/2 interactions on endothelial cells mediate their effects via representative RTKs that exert potent pro-angiogenic effects by promoting endothelial cell proliferation, survival, migration, tube formation, and protease production [23,24,25,26,30,43]. Monotherapy using small-molecule FGFR/VEGFR2 dual inhibitors is an excellent way to optimize their curative effects and expand their antitumor range [24,43]. Only a few FGFR/VEGFR inhibitors have entered into phase III clinical trials and been approved. However, as with most non-selective inhibitors, toxicity remains a significant barrier to the clinical use of non-selective small-molecule FGFRis [43]. To avoid unexpected side effects of non-selective FGFR/VEGFR inhibitors and optimize the effects of selective FGFR/VEGFR inhibitors, suitable biomarkers need to be developed to predict the efficacy of these inhibitors [24,43].

### 4.2. Selective Small-Molecule FGFRis

Selective FGFRi agents have been developed to realize on-target FGFR inhibition in patients with a/m UBC harboring FGFR abnormalities [4,5,8,21,23,24,26,30,43]. The first generation FGFR-specific TKIs aimed to target FGFR1–4 (pan-FGFR inhibitors) and included erdafitinib (JNJ42756493), rogaratinib (BAY1163877), infigratinib (BGJ398), and pemigatinib (INCB054828). The development of pan-FGFR inhibitors continues to progress towards increased selectivity and stronger binding kinetics. FGFR-selective agents have a specific toxicity profile, including hyperphosphatemia and tissue calcification due to the inhibition of FGF2/FGF3 signaling, nail toxicity, hair modifications, mucositis, retinal detachment, and muscle and joint pains. These effects are clinically manageable and reversible but can lead to discontinuation of therapy or dose reduction.

Erdafitinib (JNJ-42756493), an oral, highly selective, and reversible FGFR1-4 inhibitor, can also bind to VEGFR-2/PDGFR/CSF-1R with a lower affinity [4,5,8,21,23,24,26,30,43,46]. The antitumor activity of erdafitinib was evaluated in a phase II clinical trial in patients with a/m UBC harboring a pre-specified FGFR3 mutation or FGFR2/3 fusion [21]. In the early part of the study, patients were randomized at a 1:1 ratio to receive an intermittent or continuous dose of erdafitinib [21]. The investigator-assessed ORR was 40% (95% confidence interval (CI), 31–50) [21]. Of the 74 (49%) patients with FGFR3 mutations, 36 responded to treatment, and 4 patients of 25 (16%) with FGFR 2/3 fusion responded to treatment [21,46]. In the 22 patients who received prior immunotherapy, the response rate was 59% with erdafitinib [21,46]. After a follow up of 2 years, the median PFS was 5.5 months (95% CI, 4.2–6.0), and the median OS was 11.3 months [21,46]. The most common treatment-related adverse events (AEs) included hyperphosphatemia (77%), stomatitis (58%), diarrhea (50%), and dry mouth (46%) [21,46]. Based on these data, the U.S. FDA granted accelerated approval for erdafitinib in adult patients with a/m UBC and susceptible FGFR2/3 alterations [21,46].

The activity of rogaratinib (BAY 1163877, FGFR1-4 inhibitor) was assessed in a phase I (NCT01976741) expansion cohort of patients with a/m UBC harboring FGFR1-3 mRNA overexpression [24,43,47,48,49,50,51]. Of the evaluable patients, the ORR was 24%, and the disease control rate (DCR) was 73% [24,43]. Similar to erdafitinib (NCT02365597), ICI pre-treated patients were also ICI non-responders (9/10) but showed a higher response to rogaratinib (ORR 31%, DCR 80%) than ICI-naïve patients [24,43]. The FORT-1 study evaluated the efficacy of rogaratinib in comparison with chemotherapy (docetaxel, paclitaxel, or vinflunine) in patients with mUC who received prior cisplatin-based chemotherapy [47]. Patients were selected based on either FGFR1/FGR3 mRNA overexpression and/or FGFR-3–activating mutations or translocations [47]. On exploratory analysis in patients with FGFR3 mutations or fusions, the ORR was 52.4% for rogaratinib, and with chemotherapy, it was 26.7% [47].

Infigratinib is an oral, selective, ATP-competitive FGFR 1–3 TKI [50]. The activity of infigratinib was demonstrated in a phase I trial (NCT01004224) with a subsequent expansion cohort of 67 FGFR3-altered, a/m UBC patients, the majority of who were platinum-pretreated (59/67, 88%) [24,43]. The ORR was 25%, and the DCR was 64%, although the PFS was only 3.8 months (95% CI: 3.1–5.4 months) and the median OS was 7.8 months (95% CI: 5.7–11.6 months). The response to previous ICI was low (two of nine evaluable patients showed SD, and the remaining seven patients showed progression) [24,43]. In a phase I clinical trial, the safety and antitumor activity of infigratinib was evaluated in 132 patients with solid tumors. Based on its improved side-effect profile, a 125 mg dose given on a 3 weeks on/1 week off schedule was recommended for phase II studies [52]. In the FGFR3-mutated urothelial cohort, the ORR was 38%, and 75% achieved disease control [52]. A phase III clinical trial is currently evaluating infigratinib in patients with UBC after surgery in the adjuvant setting (NCT04197986) [53].

### 4.3. FGFR Human Monoclonal Antibodies

Monoclonal antibodies represent another class of selective inhibitors that, in the case of FGFR, function through a number of mechanisms, including the disruption of ligand binding and/or receptor dimerization or the conjugation of the antibody of interest to a cytotoxic agent (ADCs) [15,23,25,54]. Aprutumab ixadotin (BAY 1187982) is an ADC that uses a derivative of the highly potent microtubule-disrupting agent auristatin and is selective for the FGFR2-IIIb and FGFR2-IIIc isoforms. Preclinical studies showed that treatment with BAY 1187982 resulted in dose-dependent tumor regression in both triple-negative breast cancer and gastric cancer xenograft models with FGFR2 overexpression [25,55]. However, the drug was poorly tolerated, and the maximum-tolerated dose was below the estimated therapeutic threshold, resulting in the early termination of this first in-human study [25,56]. The most clinically promising FGFR2 monoclonal antibody currently in development is bemarituzumab (FPA144), which specifically targets FGFR2-IIIb and is glycoengineered to enhance antibody-dependent cell-mediated toxicity, a process whereby effector immune cells recognize and kill target cells that display the antibody [25,54,57].

MFGR1877S binds to FGFR3 with a high affinity to competitively inhibit native ligand binding and prevent receptor dimerization not only in cells with wild-type FGFR3 but also in cells with the most prevalent cancer-associated mutants of FGFR3 [58]. Phase 1 clinical trials have been completed in multiple myeloma patients with the t(4; 14) translocation causing overexpression of FGFR3 (NCT01122875) and advanced solid tumors (NCT01363024) [25,58]. MFGR1877S was well tolerated by patients in both studies, and stable disease (SD) was the best response achieved (6/14 myeloma patients and 9/26 patients in the solid tumor study, including five patients with urothelial carcinoma, two patients with adenoid cystic carcinoma, and two patients with carcinoid tumors) [58,59,60].

Vofatamab (B-701) is another selective anti-FGFR3 receptor monoclonal antibody that is being evaluated in patients with a/m UBC in a second-line setting [25,50,61]. In the preliminary analysis of 55 patients, vofatamab monotherapy (at 25 mg/kg) or in combination with docetaxel (at 75 mg/m^2^ q3w) was shown to be well tolerated. Vofatamab (B-701) was shown to be well tolerated in combination with docetaxel in patients with urothelial cell carcinoma in the FIERCE-21 study (NCT02041542). The most common side effects were decreased appetite, diarrhea, fever, asthenia, and fatigue. Not surprisingly, enhanced activity was observed in patients with FGFR3 mutations or fusions compared with patients with the wild type. However, preliminary data from the FIERCE-22 study, which combines vofatamab with the ICI, pembrolizumab, in a/m UBC, show benefit even in patients with the FGFR3 wild type compared with previous studies of pembrolizumab monotherapy (NCT03123055) [62]. Although these mAbs have shown promising antitumor effects in advanced solid tumors, their clinical potential has been only partially explored [25].

## 5. Mechanisms Underlying Therapeutic Resistance to FGFRi in a/m UBC

Primary resistance describes an initial lack of treatment response, while secondary resistance describes disease progression after an initial response to treatment and has emerged as a limiting factor in the long-term efficacy of FGFRis [24,50]. A recent review summarized various mechanisms of resistance to FGFRis, including activation of bypass signaling involving amplification or mutations in proteins appertaining to MAPK, PI3K/AKT, EGFR, PLC-γ, and STAT signaling, gatekeeper mutations conferring resistance by interfering with the binding between the receptor and the targeted agents, and intratumor heterogeneity (ITH) [23,24,25,26,30,38,39,40,41,44,50,63,64]. For example, UBC cells harboring FGFR3-TACC3 fusions acquire resistance to FGFRis through the upregulation of EGFR/HER3-dependent PI3K-AKT signaling [24,50], and mutations occurring at gatekeeper residues in FGFR, such as FGFR1 V561M and FGFR2 V565I, lead to steric hindrance within the ATP-binding pocket, which precludes the entry and binding of multiple FGFRis [24,50,63,65]. Finally, ITH, in which tumors contain different subclones and independent clones, can play a role in the treatment response [66,67].

## 6. Immune Invasion as a Potential Key Target of FGFRis in a/m UBC

A major mechanism of immune escape of cancer cells is the exhaustion of CD8+ T cells, which recognize tumor antigens [23,24,25,26,30,38,39,40,41,44]. ICIs work to restore antitumor T-cell functions [4,5,7,8,24,43]; however, the lack of existing immune cells in the TME leads to an inadequate response to monotherapy with ICIs [4,5,7,8,24,43]. ICIs seem to be less effective on the UBC TCGA luminal I subtype, based on an exploratory analysis of a phase 2 trial. The luminal I cluster had reduced expression levels of CD8+ genes, lower PD-L1 immune cell or tumor cell expression (“cold tumor”), and a lower response to the anti-PD-L1 atezolizumab [4,5,7,8,24,43]. FGFR/VEGFR dual inhibitors can reverse the TME from immunologically cold tumors into ‘hot’ tumors, leading to sensitization to ICIs [24,43].

Immunoregulatory mechanisms in a/m UBCs are clearly interconnected [1,4,8,11,38,40,41,42,43,44,45,68]. CD8+ T cells, natural killer (NK) cells, and natural killer T (NKT) cells are immune effector cells involved in tumor elimination, whereas myeloid-derived suppressor cells (MDSCs), M2-TAMs, and regulatory T (Treg) cells are immune modifier cells involved in immune evasion and tumor growth. Moreover, cancer cells can recruit immunosuppressive cells and defective dendritic cells that promote T-cell tolerance of tumor antigens. For example, MDSCs activate M2-TAMs and Tregs, but inhibit CD8+ T cells and NK cells, in part through the expression of arginase 1 (ARG1), indoleamine 2,3-dioxygenase, and inducible nitric oxide synthase (iNOS) [1,4,8,11,38,40,41,42,43,44,45,68]. In addition, MDSCs directly interact with tumor cells and promote cancer cell stemness, thereby assisting tumor maintenance, and endothelial progenitor cell-like MDSCs are involved in tumor angiogenesis. In patients with UBC, the accumulation of MDSCs correlates with advanced cancer grade, stage, and poor prognosis [1,8,11,40,42,43,44,45,68].

## 7. Rationale for Combining FGFRis and ICIs in a/m UBC

As anticancer immunity and immune tolerance in the TME are regulated by the interaction between cancer cells and immune cells, there is rationale for the application of FGFRis to target paracrine FGF signaling in the immune TME of a/m UBC. Notably, FGFRis indirectly induce the reduction or disappearance of MDSCs from the TME, partly by targeting cytokine-producing CAFs [23,24,25,26,30,38,39,40,41,44]. Furthermore, CSF-1 signaling through CSF-1R on MDSCs and TAMs is involved in the proliferation, survival, and differentiation of MDSCs and TAMs [23,24,25,26,30,38,39,40,41,44]. As CSF1 and FGF signals are both involved in the accumulation of tumor-infiltrating/promoting MDSCs and M2-TAMs, the dual inhibition of FGF and CSF1 or VEGF signaling is expected to enhance antitumor effects through the targeting of immune evasion and angiogenesis in the TME [23,24,25,26,30,38,39,40,41,44]. Finally, the aberrant tumor vasculature is another critical factor that influences the immune response in the TME. For example, VEGF, which is secreted by tumors, not only increases angiogenesis but also modulates TCR signaling to inhibit T helper type 1 and cytotoxic T-cell activity [23,24,25,26,30,38,39,40,41,44,68]. FGF/FGFR and VEGF/VEGFR inhibitors enhance T-cell recruitment by normalizing tumor blood vessels [23,24,25,26,30,38,39,40,41,44,68]. Subsequent additional vessel normalization with FGF/FGFR and VEGF/VEGFR inhibitors can lead to further activation and infiltration of effector T cells into the TME [23,24,25,26,30,38,39,40,41,44,68]. In this context, combining ICI with FGFR-targeted drugs is an appealing therapeutic option to improve the response and reduce the emergence of resistance in the management of a/m UBC.

Two tremendous recent breakthroughs in a/m UBC treatment are the approval of ICIs and the FGFRi, erdafitinib, for treating this deadly disease [4,5,7,8,23,69]. If FGFR alterations do not confer ICI resistance, and cross-resistance is low between FGFRis and ICIs, combination therapy using non-selective FGFRis (FGFR/CSF1R/VEGFR2 inhibitors) and ICIs (anti-PD-1 or anti-CTLA-4 mAb) is attractive based on their pharmacologic principles [4,5,7,8,23,24,25,26,30,38,39,40,41,44,69]. In a phase Ib/II clinical trial (NORSE study), the safety and antitumor activity of erdafitinib in combination with cetrelimab (an IgG4 anti–PD-1 inhibitor) was evaluated in patients with a/m UBC harboring susceptible FGFR2/3 alternations [70]. Patients were enrolled after progression on one or more lines of therapy, including platinum-based chemotherapy. Of the 15 patients enrolled in the study, no dose-limiting toxicities were noted; 10 patients experienced grade 3 AEs, and 3 had serious unrelated AEs, which lead to death in 2 patients. The combination of erdafitinib (8 mg with up-titration to 9 mg) with cetrelimab was deemed safe for further evaluation. In the seven patients treated with the recommended phase II dose, the ORR was 71%. This combination is currently under further evaluation in a randomized phase II clinical trial (NCT03473743).

Several trials randomizing patients between monotherapy and combination therapy are ongoing. Three trials are comparing FGFRi monotherapy to FGFRi–ICI combinations (FIDES-02 or NCT04045613, NORSE or NCT03473743, and FIGHT-205 or NCT04003610), one is comparing ICI monotherapy to FGFRi–ICI combination (FORT-2 or NCT03473756), and one is comparing FGFRis, chemotherapy, and pembrolizumab as monotherapies (THOR or NCT03390504). Selective FGFRi–ICI combination initial trials have been reported from the phase I BISCAY study, a multi-arm/multi-drug, biomarker-driven trial (NCT02546661) [71]. Module A explored AZD4547 with or without durvalumab in platinum-resistant and ICI-naïve patients with a/m UBC harboring FGFR alterations [71]. In a preliminary analysis, the AZD4547 plus durvalumab cohort showed only a modest increase in activity when compared to the AZD4547 monotherapy cohort (*n* = 21, ORR 29% versus *n* = 15, ORR 20%, respectively). The combination was overall tolerated with acceptable side-effect profiles. FIERCE-22 (NCT03123055) is a single-arm phase Ib/II study of vofatamab (fully human monoclonal antibody against FGFR3 that blocks activation of both the wild-type and genetically activated receptor, 25 mg/kg, 2-week lead-in) followed by the vofatamab–pembrolizumab combination (25 mg/kg and 200 mg, respectively, every 21 days) [62]. The study enrolled patients with advanced, platinum-resistant UBC regardless of FGFR alteration status. In a preliminary report, 28 patients had enrolled into the phase II segment (FGFR altered: *n* = 8, WT: *n* = 20) with an ORR of 40% [62]. Responses were similar between the FGFR-altered (43%) and WT (40%) cohorts. Interestingly, the translational analysis revealed that the luminal molecular subtype was associated with a higher response rate, the p53-like molecular subtype was associated with poor survival, and a lead-in vofatamab monotherapy induced inflammatory pathway alterations [62].

Lenvatinib, a multiple TKI that inhibits VEGFR1-3, FGFR1-4, PDGFRα, c-KIT, and RET [72], is a potent angiogenesis inhibitor and also an effective immunomodulator [72,73]. The dual inhibitory activity of lenvatinib against both VEGF and FGF induced broad-spectrum antitumor activity due to its antiangiogenic effects [73]. These antiangiogenetic effects convert the immunosuppressive status of the TME to a pro-tumor milieu and lead to priming of increased IFN-γ production by cytotoxic T cells [73]. Lenvatinib shows more potent antitumor activity when combined with PD-1 blockade by decreasing TAM numbers [73]. The combination of lenvatinib and pembrolizumab is being investigated as a frontline treatment in the phase III LEAP-011 trial (NCT03898180), which is evaluating the combination in cisplatin-unfit subjects with a PD-L1 combined positive score ≥10, or in patients deemed ineligible for any platinum-based regimen, regardless of PD-L1 expression [45].

## 8. Conclusions and Perspectives

FGFRis exert their antitumor activities through direct effects on tumor cells harboring FGFR alterations and through indirect effects on the TME, including the regulation of angiogenesis, immune evasion, and paracrine tumor proliferation, independent of FGFR alterations [45]. Therapeutic applications of FGFRis mark an important milestone for precision medicine in the treatment of a/m UBC. Erdafitinib was approved by FDA for use in later-line settings based on clinical activity in heavily pre-treated FGFR2/3-altered a/m UBC patients [21]. Although only approximately 20% of patients are eligible for erdafitinib, combination regimens may extend the benefit of these therapies to a larger population of patients. Since FGFR alterations may be associated with ICI resistance, FGFRi–ICI combinations may be attractive due to the potential immune-modulatory effects of FGFRis and based on the presumed non-cross-resistance of these therapeutic classes. The adverse events (AEs) related to FGFRis or ICIs as monotherapies are largely non-overlapping and can often be mitigated for both therapeutic classes with education, prompt reporting of signs/symptoms, and aggressive management (Table 2).

Despite the enthusiasm, combination FGFRi–ICI trials are mostly in the early phases of clinical development, and current clinical practice should still follow a sequential approach. To move forward with FGFRi–ICI combinations, reliable and predictive biomarkers for assessing FGFRi–ICI combinations are urgently needed to quantify the complex interplay of FGFR signaling and the immune components in the TME.

The results of ongoing trials will delineate the optimal role and sequence of FGFRi or FGFRi-based combination regimens for treating a/m UBC.

## Figures and Tables

**Table 1:** Representative FGFRi’s as single anti-cancer agents.

FGFRi	Mode of Action
Dovitinib (TKI258)	Non-selective, ATP-competitive, FGFR1-3, VEGFR1-3, PDGFR-β, FLT3, KIT inhibitor
Brivanib (BMS-540125)	Non-selective, ATP-competitive, FGFR1, VEGFR1/2, PDGFR-β inhibitor
Nintedanib (BIBF1120)	Non-selective, ATP-competitive, FGFR1-3, VEGFR1-3, PDGFR-α/β, FLT3, KIT inhibitor
Lenvatinib (E7080)	Non-selective, ATP-competitive, FGFR1-4, VEGFR1-3, PDGFR-α/β, FLT1, KIT inhibitor
Erdafitinib (JNJ-42756493)	Selective, ATP-competitive, FGFR1-4 inhibitor
Rogaratinib (BAY1163877)	
Infigratinib (BGJ398)	Selective, ATP-competitive, FGFR1-3 inhibitor
Pemigatinib (INCB054828)	
Aprutumab ixadotin (BAY 1187982)	Antibody–drug conjugates (ADCs), a fully human anti-FGFR2 monoclonal antibody conjugated by lysine side chains to a non-cleavable linker and via this an innovative auristatin W derivative (a highly potent microtubule-disrupting agent)
Bemarituzumab (FPA144)	A human monoclonal antibody specific to the splice-variant FGFR2b that inhibits binding of the ligands FGF7, FGF10, and FGF22
MFGR1877S	A human monoclonal antibody that targets FGFR3 to prevent ligand binding, receptor-receptor association, and FGFR3 signaling
Vofatamab (B-701)	A fully human monoclonal antibody against FGFR3 that blocks activation of the wildtype and genetically activated receptor

**Table 2:** Combinations of FGFRi + ICI: rationale and its applications.

Category	Rationale for Treatment Synergism between FGFRi and ICI in a/m UBC
Tumor infiltrating NK/NKT/cytotoxic CD8+ T cells	Immune effector cells involved in cancer cell elimination
Tumor infiltrating dendritic cells/MDSCs/M2-TAMs/Treg	Defective immune modifiers contributing to tumor immune evasion
MDSCs	Directly interact with tumor cells and promote cancer cell stemness Lead to immune evasion in the TME by activating M2-TAMs/Treg cells and inhibiting NK/cytotoxic CD8+ T cells
M2-TAMs	Express immunosuppressive paracrine factors, such as IL-10, TGFβ, and ARG1
Endothelial progenitor cell-like MDSCs/M2-TAM subset	Promote tumor angiogenesis
Dendritic cell-specific C-type lectin TAMs	Contribute increased levels of Treg cells/cytotoxic CD8+ T cells with an impaired cytolytic activity (reduced levels of the cytotoxins perforin, granzyme B, and IFN-γ)
Treg cells	Suppress antitumor immune activity through release of inhibitory cytokines (TGFβ, IL-10) and cell–cell contact via immune checkpoint molecules (CTLA-4, LAG3) Induce apoptosis of cytotoxic CD8+ T cells through cytolysis via perforin or granzyme, IL-2 consumption and ATP deprivation through CD38 hydrolyzing ATP to ADP and AMP
Immune exclusion phenotype caused by FGFR 3 mutations	Caused by the sequestration of cytotoxic CD8+ T cells in TME due to increased deposition of fibronectin and collagen in the extracellular matrix
ICIs	Target negative regulating cell receptors on immune cells, predominantly T cells, leading to reactivation of those cells and promotion of a durable antitumor response Seem to be less effective on UBC TCGA luminal I subtype with attenuated CD8+ cytolytic activity, lower expression of PD-L1 in both tumor cells and immune cells
FGFRis	Reverse the TME from immunologically cold tumors into hot tumors by enhancing T cell recruitment by normalizing tumor blood vessels Target immune suppressive cells in TME such as MDSCs/M2-TAMs/CAFs in direct or indirect manners

## Data Availability

Not applicable.
